# Is red blood cell distribution width a prognostic factor for colorectal cancer? A meta-analysis

**DOI:** 10.3389/fsurg.2022.945126

**Published:** 2022-10-03

**Authors:** Ze-Lin Wen, Xiong Zhou, Da-Chun Xiao

**Affiliations:** Department of Gastrointestinal Surgery, Chongqing Medical University, Yongchuan Hospital, Chongqing, China

**Keywords:** red blood cell distribution width, colorectal cancer, meta-analysis, surgery, survival

## Abstract

**Background:**

RDW might be an easy and cost-effective pre-operative prognostic factor for cancer patients. The aim of the current study was to analyze whether red blood cell distribution width (RDW) was a prognostic factor for colorectal cancer (CRC) patients who underwent radical surgery.

**Methods:**

We conducted the searching strategy in three databases including the PubMed, Embase and Cochrane Library from the inception to May 07, 2022, to find eligible studies. In this meta-analysis, we focused on the prognosis. Pooled hazard ratios (HRs) and 95% confidence intervals (CIs) were calculated for overall survival (OS), disease-free survival (DFS) and cancer-specific survival (CSS).

**Results:**

A total of seven studies involving 7,541 patients were included in this meta-analysis. After pooling up the HRs, red blood cell distribution width-coefficient of variation (RDW-CV) was not an independent prognostic factor of OS (HR = 1.48, *I*^2 ^= 90%, 95% CI = 0.93 to 2.36, *P* = 0.10), however, red blood cell distribution width-standard deviation (RDW-SD) was an independent prognostic factor of OS (HR = 1.99, *I*^2 ^= 0%, 95% CI = 1.59 to 2.49, *P* < 0.01). As for DFS, we found that RDW-CV (HR = 1.51, *I*^2 ^= 83%, 95% CI = 0.94 to 2.43, *P* = 0.09 < 0.10) and RDW-SD (HR = 1.77, *I*^2 ^= 56%, 95% CI = 0.91 to 3.43, *P* = 0.09 < 0.10) were both the independent prognostic factors. In terms of CSS, we found that RDW-CV was not an independent prognostic factor (HR = 1.23, *I*^2 ^= 95%, 95% CI = 0.72 to 2.10, *P* = 0.46).

**Conclusion:**

RDW-SD was an independent prognostic factor of OS and DFS, and RDW-CV was an independent prognostic factor of DFS.

## Introduction

The incidence of colorectal cancer (CRC) was 38.7 per 100,000 and the mortality rate was 13.9 per 100,000% (1). Among them, CRC was the third most common cancer in males and the second in females (2). The treatments of CRC include surgery, chemotherapy, radiotherapy, surgery, targeted therapy and immunotherapy (3–8). Nowadays, radical surgery is the cornerstone treatment of CRC (9, 10), which not only can treat cancer, but also help in the improvement of some comorbidities (11, 12).

Red blood cell distribution width (RDW) is a hematological parameter which can be divided into two types as follows: RDW standard deviation (RDW-SD) and RDW coefficient of variation (RDW-CV), whose unit was FL and %, respectively (13). RDW can reflect the heterogeneity of red blood cell size (14), and it has been applied to predict anemia, chronic inflammation and cardiovascular disease (15–18). Recent studies reported that RDW could predict the prognosis of patients with esophageal cancer, gastric cancer and liver cancer (19–22).

Some studies reported the relationship between RDW and CRC patients as well, however, whether RDW could affect the prognosis of CRC was controversial (13–26). Furthermore, the prognostic value of RDW-SD and RDW-CV might be inconsistent. Thus, it is necessary to analyze the exact impact of RDW (RDW-SD and RDW-CV) on CRC.

## Methods

This meta-analysis was conducted in accordance with the Preferred Reporting Items for Systematic Reviews and Meta-Analyses (PRISMA) statement (27).

### Literature search strategy

Two authors conducted the searching strategy in three databases including the PubMed, Embase and Cochrane Library independently. The searching date was May 07, 2022. As for RDW, the searching strategy included: “red blood cell distribution width” OR “red cell distribution width” OR “RDW”; As for CRC, the searching strategy included: “colorectal cancer” OR “colon cancer” OR “rectal cancer” OR “colorectal neoplasm” OR “colon neoplasm” OR “rectal neoplasm” OR “colorectal tumor” OR “colon tumor” OR “rectal tumor”. The language was limited to English and the searching scope was limited to titles and abstracts.

### Inclusion and exclusion criteria

The inclusion criteria were as follows: 1, CRC patients who underwent primary and radical surgery; 2, Pre-operative RDW (RDW-CV or RDW-SD) was tested; and 3, Overall survival (OS), disease-free survival (DFS) or cancer-specific survival (CSS) was reported. The exclusion criteria were as follows: 1, The type of article was letters, case reports, comments, reviews, or conference; 2, Repeated or overlapped data; and 3, Insufficient data reporting the prognosis including OS, DFS or CSS.

### Study selection

Two authors conducted the study selection independently. Firstly, the titles and abstracts were looked through by authors to find potentially relevant studies; Secondly, the full texts were read and discussed by the two authors based on the inclusion and exclusion criteria. If there was a disagreement, another author was due to make a final judgment.

### Data extraction

The data were extracted by two authors. The extracted article information included the first author, publishing country and publishing year. The extracted patients' data included RDW type, sample size, cut-off value of RDW, OS, DFS and CSS.

### Clinical characteristics

As for clinical-pathological characteristics, two authors collected the data independently. The third author was responsible for checking the information to ensure their accuracy and completeness. Only variables which were reported by more than two studies were allowed. The baseline characteristics included age, gender, carcinoembryonic antigen (CEA), tumor location, histological differentiation, Tumor Node Metastasis (TNM) stage, vascular invasion, and adjuvant chemotherapy.

### Quality assessment

The Newcastle-Ottawa Scale (NOS) was used to evaluate the quality of the included studies (28). The score equaled 9 points represented high quality, the score equaled 7 or 8 points represented medium-quality and the score which was less than 7 points represented low quality.

### Statistical analysis

In this meta-analysis, we focused on the prognosis. Pooled hazard ratios (HRs) and 95% confidence intervals (CIs) were calculated for OS, DFS and CSS. The *I*^2^ value and the results of the chi-squared test were used to assess the statistical heterogeneity (29, 30). High heterogeneity was considered when *I*^2^>50%; in such cases, the random effects model was used, and *P* < 0.1 was considered statistically significant. The fixed effects model was used when *I*^2^≤50%, and *P* < 0.05 was considered statistically significant. This meta-analysis was performed with RevMan 5.3 (The Cochrane Collaboration, London, United Kingdom).

## Results

### Study selection

**Figure 1:**
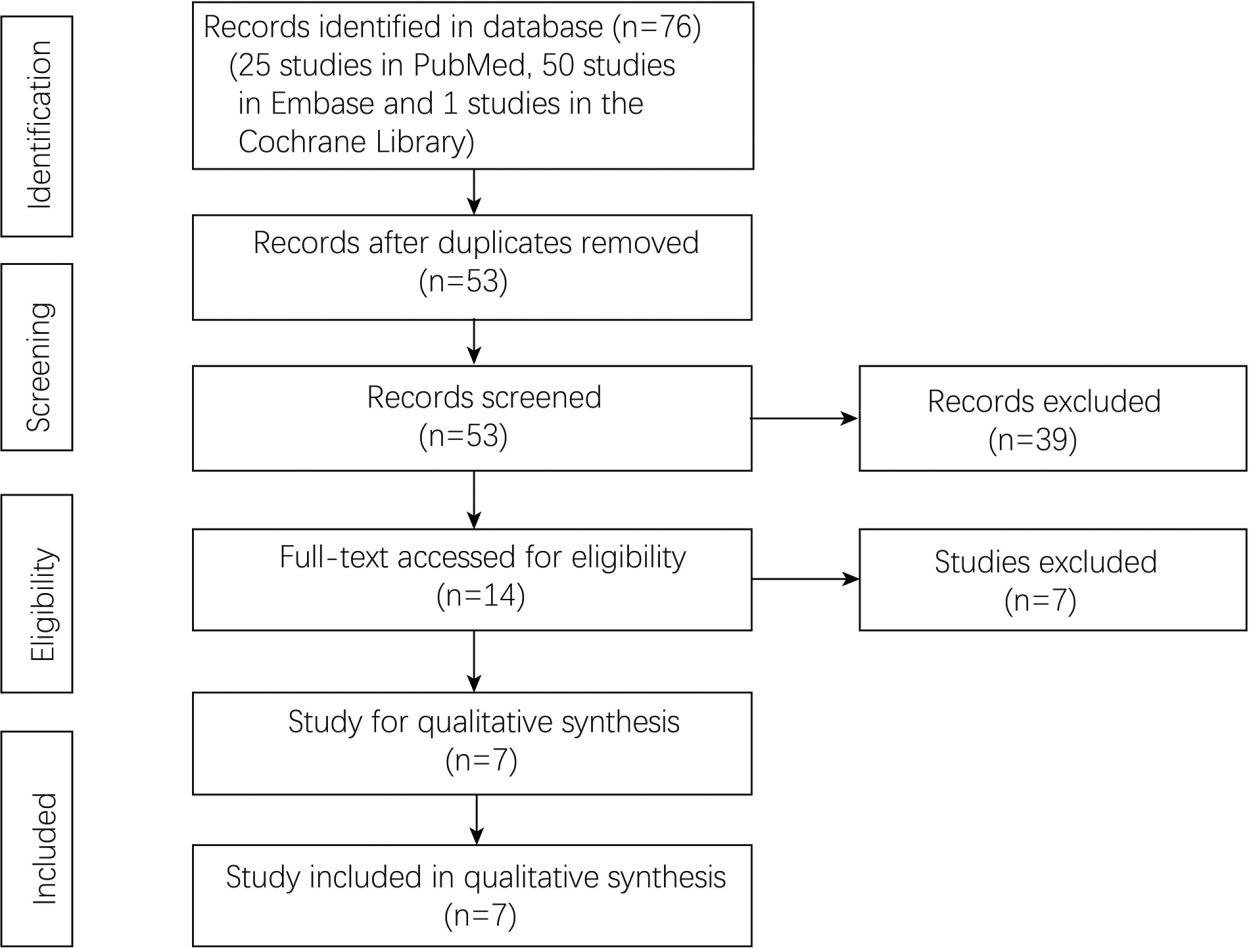
Flowchart of study selection.

A total of 76 studies were found in the databases, including 25 studies in the PubMed, 50 studies in the Embase and 1 study in the Cochrane Library. Finally, seven studies (23–26, 31–33) were included for final analysis. The flow chart of the study selection was shown in Figure 1.

### Baseline characteristics

Seven studies included 7,541 patients were included in this meta-analysis. The publication year ranged from 2018 to 2022. Two studies were from China, two studies were from Japan, one study was from Italy, one study was from United Kingdom and one study was from Switzerland. The study date was from 2001 to 2020. Three studies reported RDW-SD and five studies reported RDW-CV. The cut-off values and NOS were shown in Table 1.

**Table 1:** Baseline characteristics of included studies.

Author	Year	Country	Study date	Patients	RDW type	Sample size	Cut-off volume	NOS
Ide S	2020	Japan	2001–2017	RC	RDW-SD	120	47.1 fl	7
Pedrazzani C	2020	Italy	2005–2016	CRC	RDW-CV	591	14.1%	8
McSorley ST	2019	United Kingdom	2008–2017	CRC	RDW-CV	824	NA	8
Chen WC	2022	China	2016–2019	CRC	RDW-SD	143	12.6 fl	7
Sato R	2022	Japan	2013–2020	CRC	RDW-CV	85	13.8%	7
Cheng KC	2022	Switzerland	2004–2018	CRC	RDW-CV	5153	13.8%	8
Zhang XB	2018	China	2009–2014	RC	RDW-CV/RDW-SD	625	14.1%/48.2 fl	8

Abbreviations: RDW, red blood cell distribution width; NA, not applicable; NOS, Newcastle-Ottawa Scales; RC, rectal cancer; CRC, colorectal cancer.

### Clinical characteristics

After pooling up the odds ratio and 95% CI, there were more older patients, higher CEA level, and more TNM stage II in the high RDW group than in the low RDW group. Other characteristics including gender, tumor location, histological differentiation, TNM stage III, vascular invasion, and adjuvant chemotherapy were not significantly different between the two groups (Table 2).

**Table 2:** Summary of characteristics between high RDW group and Low RDW group.

Characteristics	Studies	Participants (High RDW/Low RDW)	Odds Ratio [95% CI]	Model	Heterogeneity
Age					
Younger	2	312/398	Reference	Reference	Reference
Older	2	312/398	2.13 [1.57, 2.90]; *P* = 0.00	FE	*I*^2^ = 0.00%; *P* = 0.93
Gender					
Female	3	2496/3367	Reference	Reference	Reference
Male	3	2496/3367	1.02 [0.42, 2.51]; *P* = 0.96	RE	*I*^2^ = 95.38%; *P* = 0.00
CEA					
<5	3	1755/2300	Reference	Reference	Reference
≥5	3	1755/2300	1.60 [1.39, 1.85]; *P* = 0.00	FE	*I*^2^ = 0.00%; *P* = 0.90
Tumor location					
Right colon	2	1201/2988	Reference	Reference	Reference
Left colon	2	1201/2988	0.56 [0.31, 1.02]; *P* = 0.06	FE	*I*^2^ = 47.80%; *P* = 0.17
Histological differentiation					
Well or moderate	3	2244/2988	Reference	Reference	Reference
*P*oor	3	2244/2988	1.37 [0.83, 2.26]; *P* = 0.22	FE	*I*^2^ = 12.59%; *P* = 0.32
TNM stage					
I	2	2449/3329	Reference	Reference	Reference
II	2	2449/3329	2.20 [1.68, 2.87]; *P* = 0.00	FE	*I*^2^ = 47.82%; *P* = 0.17
III	2	2449/3329	1.39 [0.94, 2.07]; *P* = 0.10	FE	*I*^2^ = 0.00%; *P* = 0.80
Vascular invasion	2	312/398	0.71 [0.28, 1.78]; *P* = 0.47	RE	*I*^2^ = 60.80%; *P* = 0.11
Adjuvant chemotherapy	2	312/398	2.10 [0.74, 6.01]; *P* = 0.17	RE	*I*^2^ = 81.24%; *P* = 0.02

Abbreviations: RDW, red blood cell distribution width; CI, confidence intervals; CEA, carcinoembryonic antigen; TNM, Tumor Node Metastasis.

### OS of RDW

**Figure 2:**
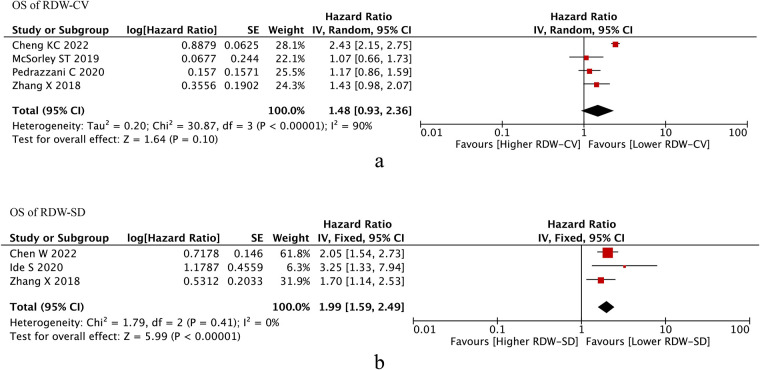
Os of RDW. (**A**) OS of RDW-CV; (**B**) OS od RDW-SD. Abbreviations: OS, overall survival; RDW, red blood cell distribution width.

Four studies reported OS of RDW-CV, after pooling up the HRs, RDW-CV was not an independent prognostic factor of OS (HR = 1.48, *I*^2 ^= 90%, 95% CI = 0.93 to 2.36, *P* = 0.10) (Figure 2a).

Three studies reported OS of RDW-SD, after pooling up the HRs, RDW-CV was an independent prognostic factor of OS (HR = 1.99, *I*^2 ^= 0%, 95% CI = 1.59 to 2.49, *P* < 0.01) (Figure 2b).

### DFS of RDW

**Figure 3:**
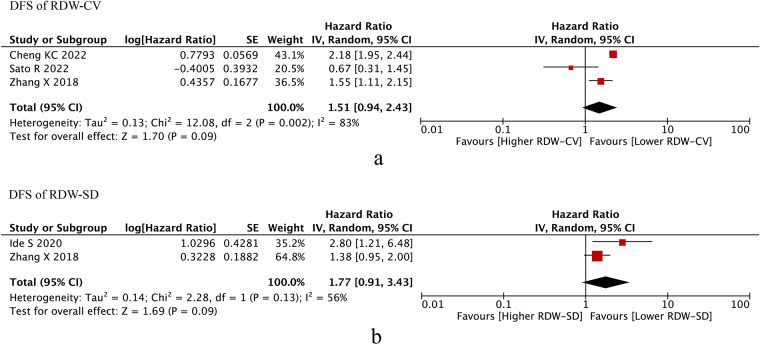
DFS of RDW. (**A**) DFS of RDW-CV; (**B**) DFS od RDW-SD. Abbreviations: DFS, disease-free survival; RDW, red blood cell distribution width.

Then, we conducted meta-analysis of RDW (RDW-CV/RDW-SD) on DFS. We found that RDW-CV (HR = 1.51, *I*^2 ^= 83%, 95% CI = 0.94 to 2.43, *P* = 0.09 < 0.10) and RDW-SD (HR = 1.77, *I*^2 ^= 56%, 95% CI = 0.91 to 3.43, *P* = 0.09 < 0.10) were both independent prognostic factors of DFS (Figures 3A,B).

### CSS of RDW

**Figure 4:**
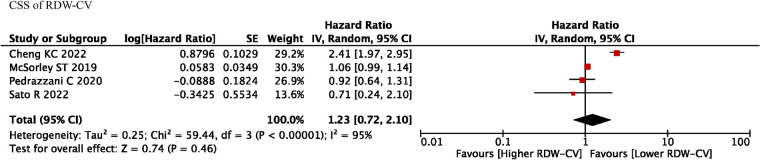
CSS of RDW-CV. Abbreviations: CSS, cancer-specific survival; RDW, red blood cell distribution width.

Four studies reported RDW-CV on the prognostic roles on CSS, and we found that RDW-CV was not an independent prognostic factor (HR = 1.23, *I*^2 ^= 95%, 95% CI = 0.72 to 2.10, *P* = 0.46) (Figure 4). However, no information was found about RDW-SD on the prognostic roles on CSS.

### Sensitivity analysis

Repeated meta-analysis was performed by excluding one study at a time, and the exclusion of any one study did not significantly alter the results.

## Discussion

A total of seven studies involving 7541 patients were included in this meta-analysis. After pooling up the HRs, RDW-CV was not an independent prognostic factor of OS, however, RDW-SD was an independent prognostic factor of OS. As for DFS, we found that RDW-CV and RDW-SD were both independent prognostic factors. In terms of CSS, we found that RDW-CV was not an independent prognostic factor. As for clinical characteristics, the high RDW group had more older patients, higher CEA level, and more TNM stage II than the low RDW group.

RDW can reflect the heterogeneity of red blood cell size (14), and the primary role of RDW is to diagnose anemia (13). The increase of RDW could accompanied by other cancer prognostic risk factors including age, later TNM stage and higher tumor markers level (34, 35). Furthermore, RDW is also associated with various diseases such as heart disease, lung disease, and even trauma (14, 36). In addition, RDW is also considered as an indicator for some inflammatory diseases including pancreatitis and hepatitis (35, 36). However, the mechanism has not been clearly demonstrated.

Previous studies had reported the relationship between RDW and the prognosis of CRC (23–26, 31–33). Zhang X et al. (23) reported that elevated RDW could be an independent factor for non-metastatic rectal cancer; Cheng KC et al. (37) analyzed 5,315 CRC patients and did propensity score matching analysis, they found that RDW was a predictor of OS, DFS and CSS. However, Pedrazzani C et al. (25) reported that RDW did not seem to influence OS or CSS, independently. Moreover, McSorley ST et al. (26) reported the same results that RDW was not a predictor of prognosis. Therefore, it is necessary to analyze the exact impact of RDW on CRC (38).

There were many factors which could affect the prognosis of CRC, including tumor stage, tumor size, age, body mass index (BMI), type 2 diabetes mellitus and so on (39–44). Prognostic indicators related to blood examination included lymphocyte count ratio (NLR), platelet count and lymphocyte count ratio (PLR), etc (31, 45, 46). The main reason that NLR and PLR could affect the prognosis was that they were important markers of systemic inflammation (23,24). Furthermore, PLR and NLR levels increased the body's inflammatory response, promoted tissue infiltration and angiogenesis (47). Similarly, in our meta-analysis, RDW could also affect the prognosis of CRC, the mechanism might be that RDW was another important marker of systemic inflammation as well.

Besides the systemic inflammation mechanism, RDW was thought to reflect oxidative stress, malnutrition, dyslipidemia, hypertension, erythrocyte fragmentation and erythropoietin alterations (48). Furthermore, RDW correlated with plasma markers of inflammation, such as high-sensitivity C-reactive protein (hs-CRP) values and erythrocyte sedimentation rate (ESR) (49). RDW was shown to reflect increased levels of circulating cytokines, including interleukin 6 (IL-6) and tumor necrosis factor-alpha (TNF-α) (50). Thus, these findings suggested that increased RDW might reflect inflammatory responses, malnutrition status and elevated oxidative stress, leading to the hypothesis that RDW was associated with poorer prognosis.

To our knowledge, previous studies had controversy about the effect of RDW on the prognosis of CRC, and this is the first study pooling up all the data to identify the accurate prognostic roles of RDW on CRC patients. Some limitations existed in this study. First, we included seven studies whose sample size was relatively small; Second, the cut-off of RDW-CV and RDW-SD was inconstant, which might cause inaccuracy; Third, small number of studies reporting OS, DFS and CSS, therefore, heterogeneity occurred, random-effects test was adopted.

In conclusion, RDW-SD was an independent prognostic factor of OS and DFS, and RDW-CV was an independent prognostic factor of DFS.

## Data Availability

The original contributions presented in the study are included in the article/Supplementary Material, further inquiries can be directed to the corresponding author/s.
